# High throughput sequencing of two celery varieties small RNAs identifies microRNAs involved in temperature stress response

**DOI:** 10.1186/1471-2164-15-242

**Published:** 2014-03-27

**Authors:** Meng-Yao Li, Feng Wang, Zhi-Sheng Xu, Qian Jiang, Jing Ma, Guo-Fei Tan, Ai-Sheng Xiong

**Affiliations:** 1 State Key Laboratory of Crop Genetics and Germplasm Enhancement, Ministry of Agriculture Key Laboratory of Biology and Germplasm Enhancement of Horticultural Crops in East China; College of Horticulture, Nanjing Agricultural University, Nanjing, People’s Republic China

**Keywords:** microRNAs, Temperature stress, Deep sequencing, qRT-PCR, Celery

## Abstract

**Background:**

MicroRNAs (miRNAs) are small, non-coding RNAs of 20 to 24 nucleotides that regulate gene expression and responses to biotic and abiotic stress. Till now, no reports have previously been published concerning miRNAs in celery.

**Results:**

Two small RNAs libraries were constructed from two celery varieties, ‘Jinnan Shiqin’ and ‘Ventura’, and characterized by deep sequencing. A total of 431 (418 known and 13 novel) and 346 (341 known and five novel) miRNAs were identified in celery varieties ‘Jinnan Shiqin’ and ‘Ventura’, respectively. Potential miRNA-target genes were predicted and annotated by screening diverse protein databases, including Gene Ontology, Cluster of Orthologous Groups and Kyoto Encyclopedia of Genes and Genomes. Significant differential expression between the two varieties was seen for 221 miRNAs. qRT-PCR was used to analyze the abundance of six miRNAs under cold and heat stress conditions. The results showed that miRNAs may have important functions in controlling temperature stress in celery.

**Conclusion:**

A large number of miRNAs were identified in celery, and their target genes, functional annotations, and gene expression patterns have been explored.

These findings provide the first information on celery miRNAs and enhance understanding of celery miRNA regulatory mechanisms under extreme temperature stress.

## Background

MicroRNAs (miRNAs) are small, non-coding RNAs found in animals and plants, and mainly function in regulating gene expression at the post-transcriptional level [1]. miRNAs are highly conserved in eukaryotes, and are an important component in the evolution of genetic regulation [2,3]. Mature miRNAs are generated from primary miRNAs (pri-miRNAs) via two steps. The pri-miRNAs are cut by RNA polymerase II into precursor miRNAs (pre-miRNAs) that have a stem-loop structure and are 70 to 100 nucleotides (nt) in length. The pre-miRNAs are then cleaved into mature miRNAs of 19 to 23 nt by Dicer-Like1 in the cytoplasm [4].

The first two characterized miRNAs, lin-4 and let-7, were identified as regulators of the juvenile-to-adult phase transition in *Caenorhabditis elegans*[5,6]. The latest miRBase database (miRBase19) contains 21,264 entries representing hairpin pre-miRNAs, expressing 25,141 mature miRNA products in 193 species. Numerous studies have revealed that miRNAs are involved in diverse biological and metabolic processes, such as regulation of cell growth and development, abiotic stress response, pathogen defense and gene translational repression [7-10]. Levels of miR156, miR167, and miR164 increase during virus intrusion [11,12]. Furthermore, miR172 and miR159 affect flowering time regulation in plants [13,14], whereas miR160 has important roles in the regulation of plant development and hormonal signals [15]. In cold conditions, miR393 over-expression is induced [16].

Celery (*Apium graveolens* L.) is an annual or perennial herb belonging to the *Apiaceae* family. Although celery originated from the Mediterranean and the Middle East, it is now cultivated worldwide. Celery is rich in carotene, vitamins, carbohydrates, and volatile aroma compounds, and has excellent medicinal properties to regulate the digestive system and blood lipids [17-19]. However, celery is one of the most common allergenic foods in many European countries [20]. Numerous allergenic compounds contained in celery tissue can induce allergic symptoms [21]. miRNAs have significant biological functions in metabolic and immune responses [8,22], and exploring the miRNAs present in celery will provide a foundation for the study of their functions. Till now, there have been no previously published reports concerning miRNA s in *Apiaceae* species.

Extreme temperature is one of the major limiting factors on celery growth and yield. Low temperatures can lead to early bolting, and high temperatures can induce various diseases. To investigate the roles of celery miRNAs under temperature stress, two varieties, ‘Jinnan Shiqin’ and ‘Ventura’, were selected for small RNA and transcriptome sequencing. ‘Jinnan Shiqin’ was bred by the Institute of Tianjin City, China, while ‘Ventura’ originated in the United States and was introduced to China. These two varieties have similar phenotypes, although they originated from different geographical areas. Here, the significant differential expression miRNAs were detected between two varieties, and the results provide useful information for miRNA that can response to temperature stress.

## Results

### Sequence analysis of small RNAs

**Figure 1:**
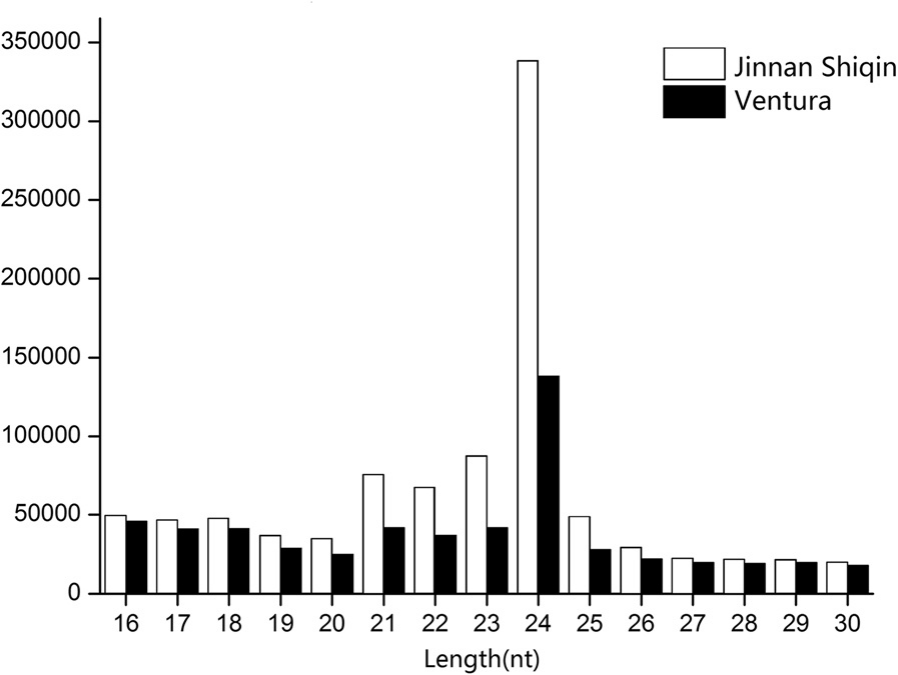
Length distributions of small RNAs in two celery varieties.

To identify the low-abundance candidate miRNAs in the ‘Jinnan Shiqin’ and ‘Ventura’ celery varieties, Solexa sequencing was performed on the small RNAs libraries. A total of 6,951,840 and 6,057,911 reads were generated from ‘Jinnan Shiqin’ and ‘Ventura’ libraries, respectively. After filtering the reads for those of low quality, 3’ insert null, poly(A), length < 16 or length > 30, the majority of small RNAs were 21 to 24 nt in length (Figure 1). The length distributions of small RNAs were similar for both varieties. The 24 nt small RNAs were the most abundant, representing 35.75% and 24.50% of small RNAs in ‘Jinnan Shiqin’ and ‘Ventura’, respectively. This result is consistent with reports for other plants, such as *Arabidopsis thaliana*, trifoliate oranges, peanuts and olives [23-26]. Some studies have reported that 24 nt siRNAs are involved in heterochromatin modification, particularly in genomes with high repetitive sequence content [27,28].

The small RNAs were further classified into different categories by performing BLAST searches against Rfam. Non-coding RNAs included miRNA, rRNA, tRNA, snoRNA, snRNA, and other unannotated RNAs. More categories of small RNAs were present in ‘Jinnan Shiqin’ than in ‘Ventura’ (Table 1).

**Table 1:** Distribution of small RNAs among different categories in ‘Jinnan Shiqin’ and ‘Ventura’

**Category**	**‘Jinnan Shiqin’**		**‘Ventura’**	
	**Unique tags**	**Total tags**	**Unique tags**	**Total tags**
miRNA	431	470923	346	109348
rRNA	83264	786363	64745	898835
tRNA	17811	124663	13497	120933
snoRNA	12647	36493	11176	29581
snRNA	860	3121	801	2848
Unannotated	59714	212201	53110	197038
Total	1742714	1633764	143670	1358583

### Identification of conserved miRNAs

**Figure 2:**
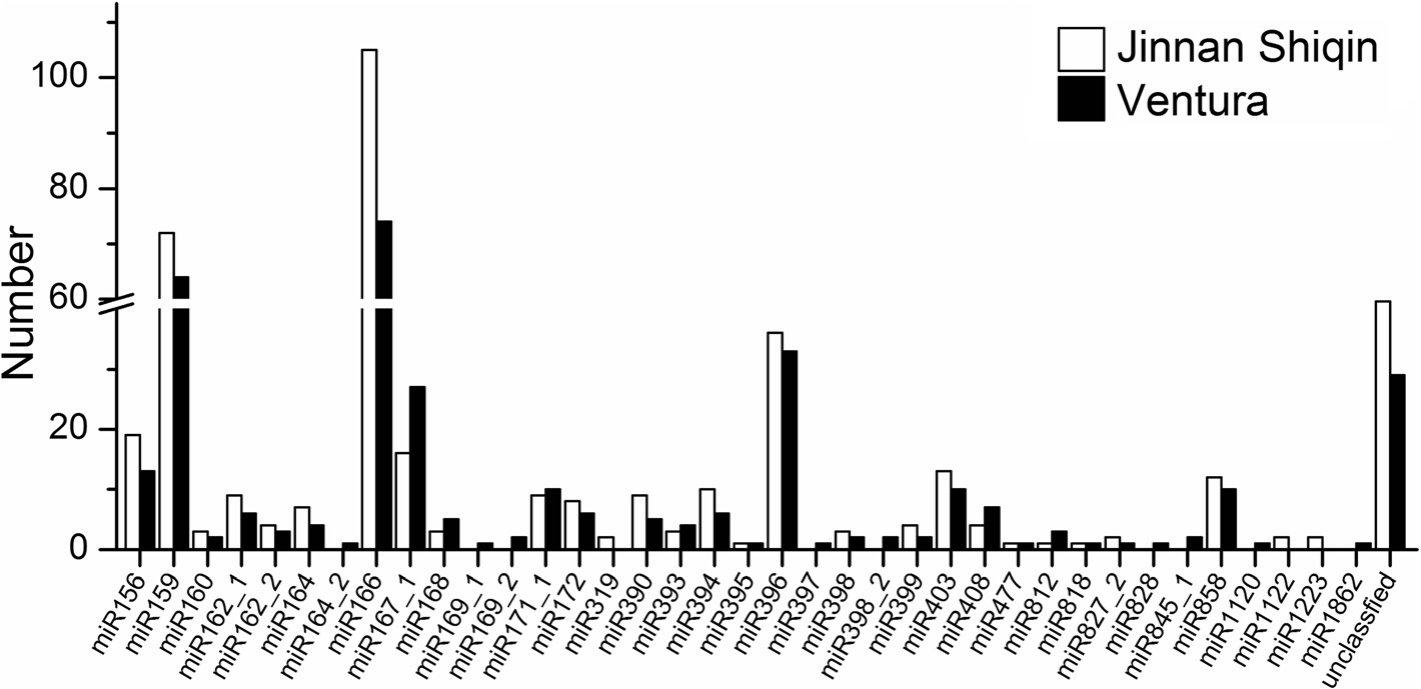
Numbers of identical miRNA members in each family in ‘Jinnan Shiqin’ and ‘Ventura’.

**Figure 3:**
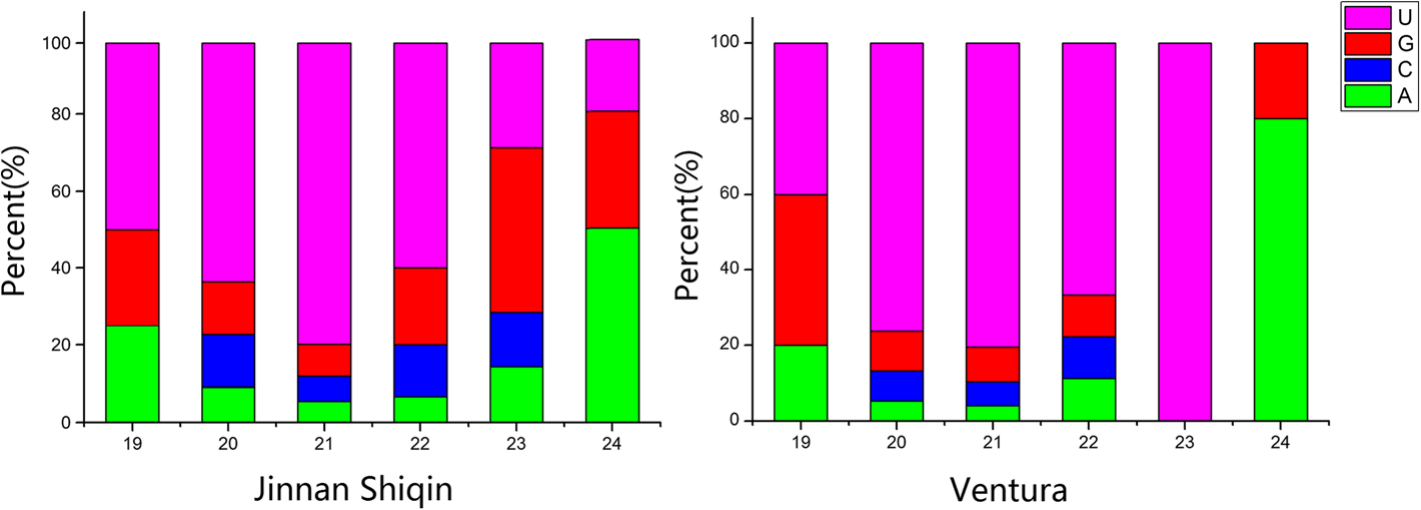
First nucleotide bias of miRNAs in ‘Jinnan Shiqin’ and ‘Ventura’.

To identify conserved miRNAs in the two celery varieties, small RNA sequences were compared with currently known mature plant miRNAs in the miRBase. Total of 418 unique miRNA sequences belonging to 28 miRNA families were identified in ‘Jinnan Shiqin’, and total of 341 unique miRNA sequences belonging to 34 miRNA families were identified in ‘Ventura’ (Figure 2). Most of the identified miRNAs showed high sequence similarities to miRNAs in other plants. The distributions of miRNA families were similar between the two libraries. Several families, such as miR156, miR159, miR166, miR167-1 and miR396, were relatively abundant, whereas some families were not. The length of miRNAs and nucleotide preference distributions are shown in Figure 3. The majority of miRNAs tended to start with 5’-U and not with 5’-G, which is consistent with typical miRNA sequence patterns.

### Identification of novel miRNAs

To identify novel miRNA sequences, all unannotated small RNAs were searched against transcriptome sequence data (SRX326597). After searching for potential pre-miRNAs and predicting their hairpin-like structures, 13 and five unique sequences were identified as novel miRNAs in ‘Jinnan Shiqin’ and ‘Ventura’, respectively (Table 2 and Table 3). Only one novel miRNA, celery-miR-27, was identified in both celery varieties. The novel miRNA sequences were 20 to 24 nt in length, and 24 nt reads were the most abundant. The range in length of pre-miRNAs was 77 to 102 nt in ‘Jinnan Shiqin’, with an average of 94 nt. The average minimum free energy values in ‘Jinnan Shiqin’ and ‘Ventura’ were −23.18 and −31.12 kcal/mol, respectively, which are much higher than values for other plant pre-miRNAs (−59.5 and −71.0 kcal/mol in *Arabidopsis* and rice, respectively) [29].

**Table 2:** Novel miRNA candidates in ‘Jinnan Shiqin’

**miRNA ID**	**Sequence (5′-3′)**	**Length(nt)**	**Start/end precursor**	**Length of precursors(nt)**	**Arm**	**MFE (kcal/mol)**
celery-miR-1	AAAATCGCTAGGCGGACCAGGGCG	24	66-166	101	3′	−19.36
celery-miR-4	ACAGGGGTCCATCACTATTAATAA	23	535-629	95	5′	−19.43
celery-miR-5	ACGAATTTAAAGATATTAACAATC	22	263-363	101	5′	−18.90
celery-miR-7	ACTGTAGTTTTCATTGGTTTTAC	23	1903-1999	97	5′	−20.40
celery-miR-9	AGTGATTGATCTGTTTTGAT	20	207-293	87	3′	−23.90
celery-miR-14	CGGAACCAGGATTTTTGATGTCC	23	93-194	102	5′	−30.10
celery-miR-16	GAACGGCGTCGGACGGAGCCGCCC	24	499-585	87	3′	−26.40
celery-miR-17	GCGGGCTGATCCGGGTTGAAGACA	24	13-107	95	3′	−24.70
celery-miR-19	GTTGTTATGTATTCGTTGTTATGA	24	551-645	95	3′	−19.43
celery-miR-20	GTTTTACTGCTAGAAGAAATGA	22	3431-3507	77	5′	−21.00
celery-miR-21	TAATGCTCTTCGTACTGCATC	21	1089-1186	98	3′	−33.50
celery-miR-24	TCCGGATTCTCCTTTTTTTCGAAC	24	9-99	91	3′	−19.30
celery-miR-27	TTTAGCTTGAACTATAGATT	20	1522-1617	96	3′	−24.90

**Table 3:** Novel miRNA candidates in ‘Ventura’

**miRNA ID**	**Sequence (5′-3′)**	**Length(nt)**	**Start/end precursor**	**Length of precursors(nt)**	**Arm**	**MFE (kcal/mol)**
celery-miR-6	ACGGAACCAGGATTTTTGATGTCC	24	95-195	101	5′	−30.30
celery-miR-13	ATTTGCTGTTAACGGGGTTCGAAC	24	213-301	89	3′	−27.90
celery-miR-18	GTTAGTAGAAACAGATCCAAT	21	392-489	98	3′	−45.20
celery-miR-22	TAGGAAGACTGTTCTCAGTGA	21	82-170	89	5′	−27.30
celery-miR-27	TTTAGCTTGAACTATAGATT	20	1522-1617	96	3′	−24.90

### Prediction and annotation of miRNA target genes

**Figure 4:**
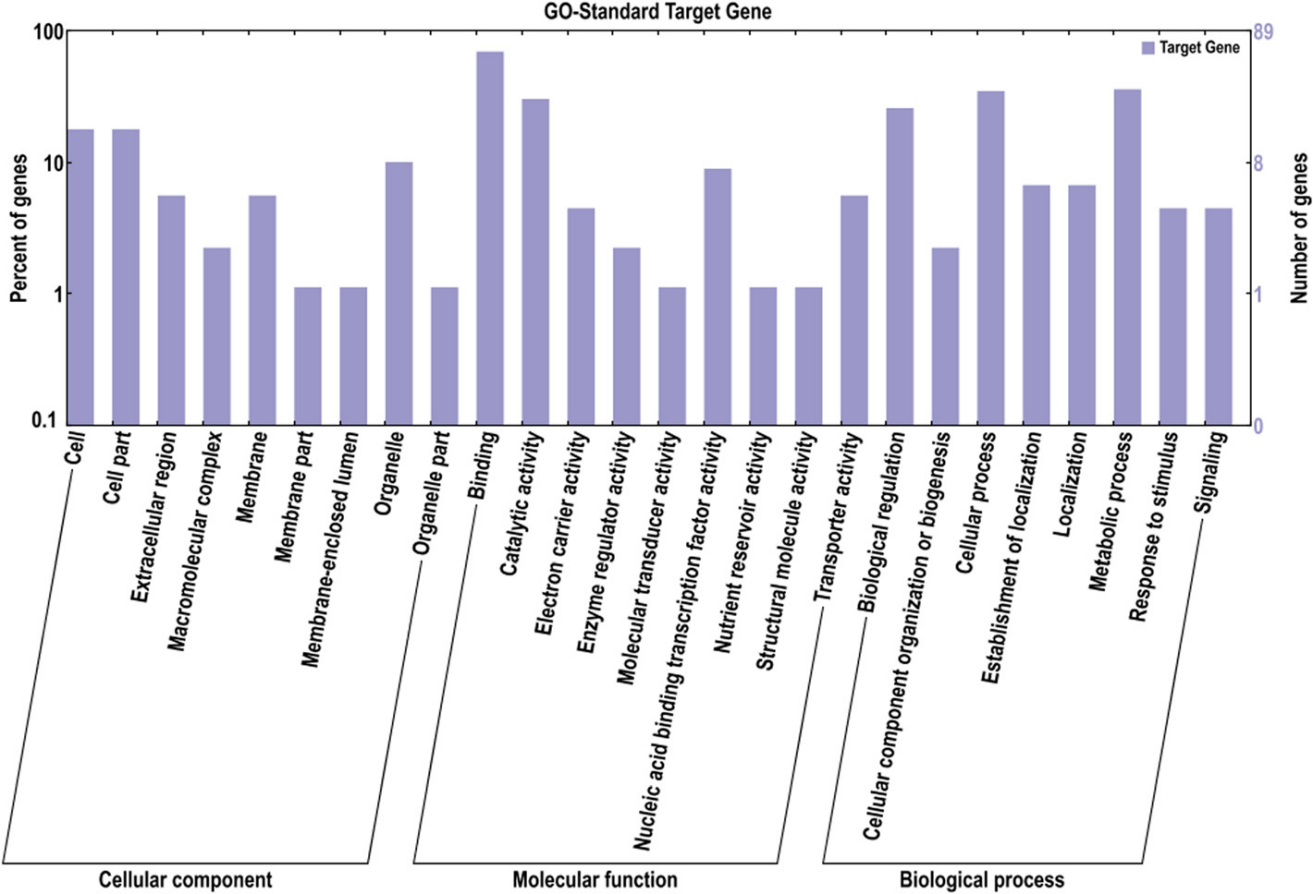
GO categories and distribution of miRNA targets in celery.

**Figure 5:**
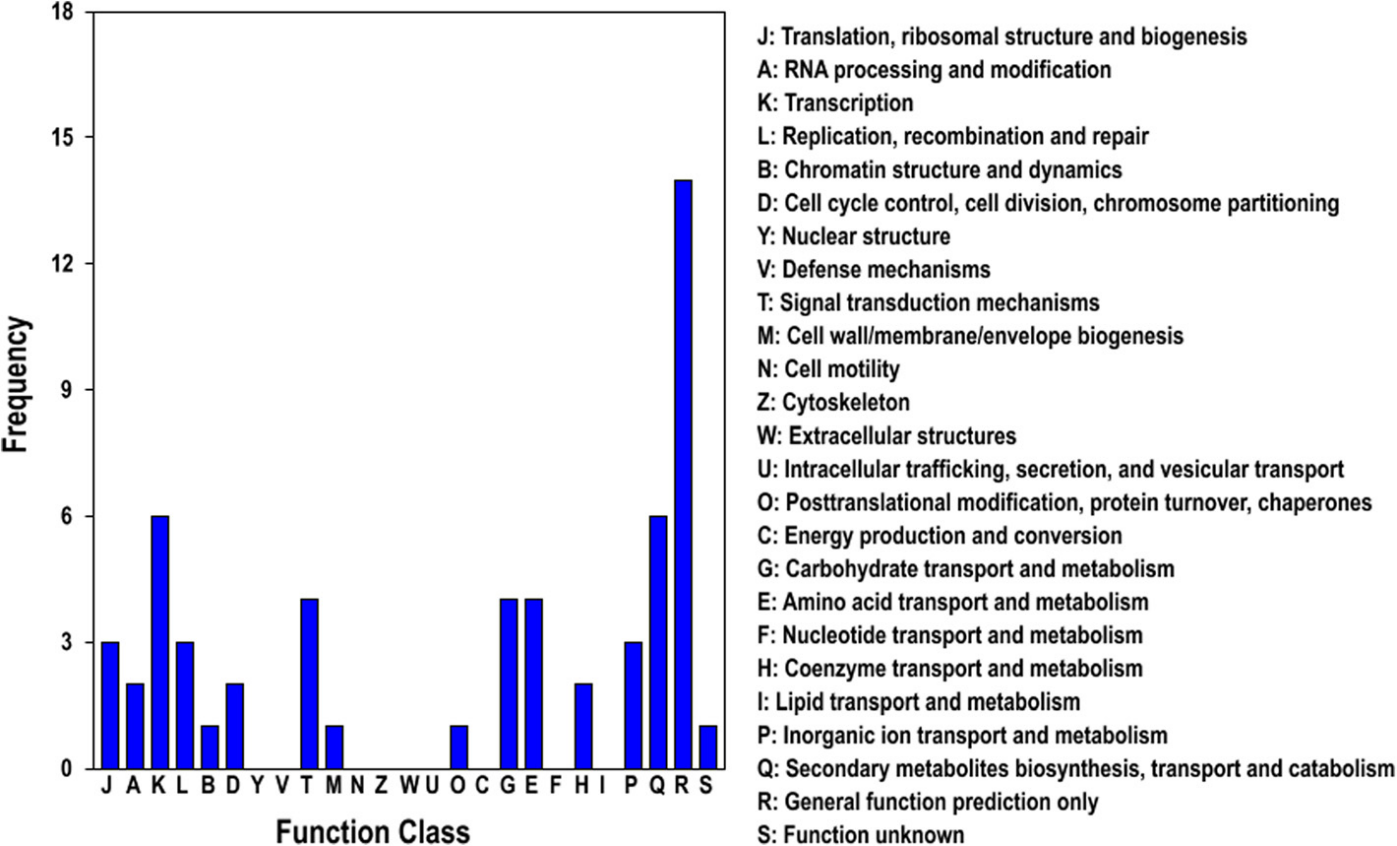
COG function classification of consensus sequences in celery.

To better understand the functions of the identified miRNAs, putative target genes were predicted using the psRNA Target program. A total of 503 and 408 potential target genes were identified in ‘Jinnan Shiqin’ and ‘Ventura’, respectively. To evaluate the potential functions of these miRNA target genes, GO analysis was used [30]. The miRNA target genes were categorized according to biological process, cellular component, and molecular function (Figure 4). Fifty-six genes were categorized as cellular components. Based on molecular function, 111 genes were classified into nine categories, among which the most over-represented GO terms were binding and catalytic activities. Eight different biological processes were found, among which the three most frequent terms were metabolic process, cellular process, and biological regulation. To further evaluate the completeness of the transcriptome and the effectiveness of the annotation process, the annotated sequences were searched for genes that could be assigned to Clusters of Orthologous Groups (COG) classifications [31]. Overall, 57 sequences were assigned to COG classifications (Figure 5), and COG-annotated putative proteins were functionally classified into at least 25 molecular families. The cluster for general function prediction was the largest group, followed in descending order by translation, secondary metabolite biosynthesis, and signal transduction. The biological interpretation of these target genes was further examined using KEGG pathway analysis. Twenty-six different pathways were found, and the most frequently represented pathways included those for transcription factors (ten enzymes), plant hormone signal transduction (four enzymes), chromosomes (four enzymes), and protein kinase inhibitors (3 enzymes).

### Differential expression of miRNAs between celery varieties

**Figure 6:**
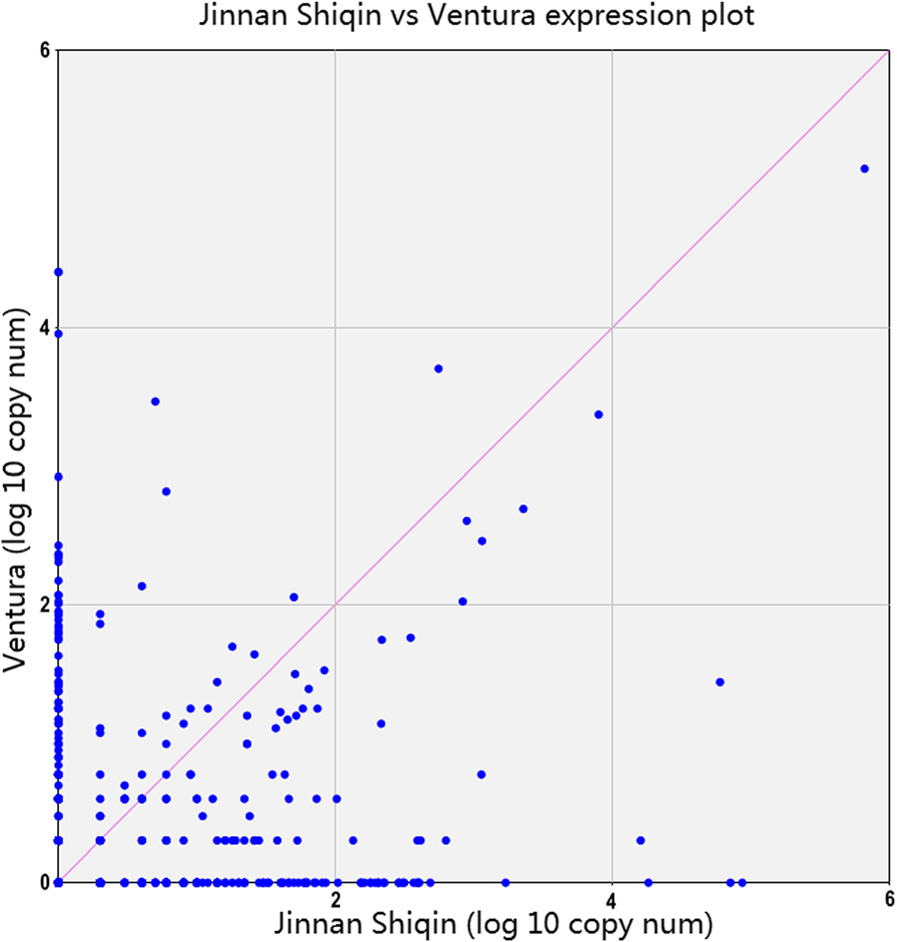
Expression distribution of miRNAs between ‘Jinnan Shiqin’ and ‘Ventura’.

The differences between the miRNA profiles for the two celery varieties are possibly related to differences in their responses to the surrounding environment. IDEG6 software was used to analyze the miRNAs of both varieties. A false discovery rate (FDR) < 0.001 and an absolute threshold value of the log2 ratio fold-change > 1 were used to determine the statistical significance of miRNA abundance. A total of 221 differentially expressed miRNAs were selected from the two libraries (Figure 6 and Additional file 1: Table S1). In ‘Jinnan Shiqin’, 55 genes were up-regulated, whereas 166 genes were down-regulated. Some families showed large differences in abundance between the two varieties. For example, mtr-miR166e and osa-miR396a were more abundant in ‘Jinnan Shiqin’, whereas sbi-miR166b and ghr-miR396b were more abundant in ‘Ventura’.

### qRT-PCR analysis of miRNAs following cold or heat stress

**Figure 7:**
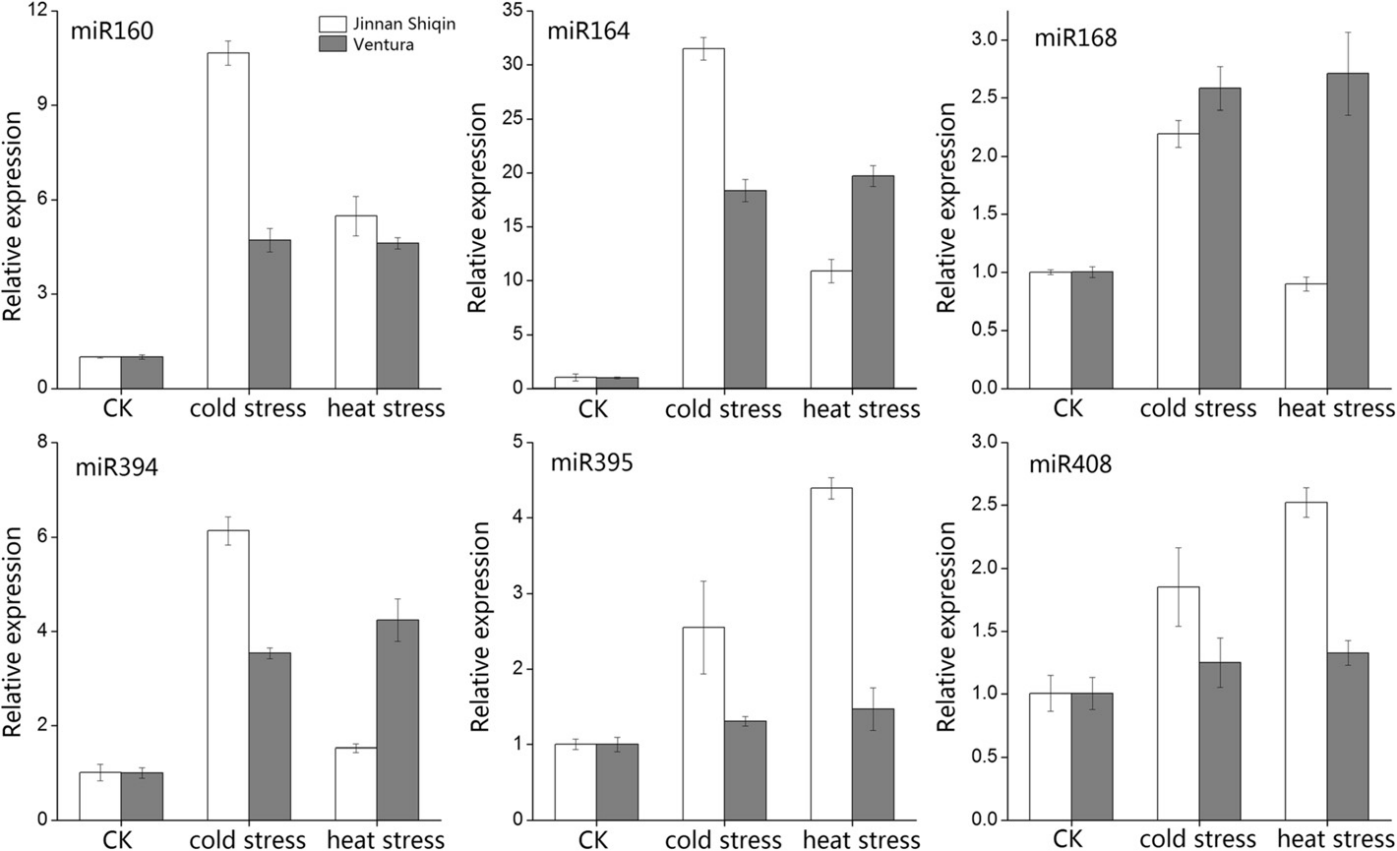
**qRT-PCR analysis of miRNAs in ‘Jinnan Shiqin’ and ‘Ventura’.** CK: control check. Error bars were calculated based on three replicates.

In this study, six miRNAs (miR160, miR164, miR168, miR394, miR395 and miR408), whose expression was previously reported to be regulated by cold or heat stress, were analyzed after temperature treatment. The changed profiles of these miRNAs are shown in Figure 7. Almost all of these miRNAs were up-regulated by cold or heat stress in both celery varieties. Aside from miR168, the abundance of the miRNAs was higher under cold stress in ‘Jinnan Shiqin’ than in ‘Ventura’, with the biggest change in miR164. After heat treatment, miR395 and miR408 were highly abundant in ‘Jinnan Shiqin’, whereas miR164, miR168, and miR394 were more abundant in ‘Ventura’. These results indicate that these six miRNAs are involved in temperature stress responses during a relatively short period of time.

## Discussion

High throughput sequencing technology has been extensively applied in small RNA research. Thousands of miRNAs and their functions have been identified in higher plants. To date, only a single report detailing transcriptome data in celery has been published, and thus, comprehensive miRNA information is not available for this plant [32]. Moreover, there are no reports concerning miRNAs in other species of *Apiaceae*. In the present study, miRNAs were identified and characterized from two celery varieties, namely, ‘Jinnan Shiqin’ and ‘Ventura’, which come from different geographical sources but have similar morphology. Till now, this study is the first to identify and investigate small RNAs in celery, and the results provide new information for further research into the functions, biological pathways and evolution of target genes related to temperature stresses in celery.

Over six million reads of 16 to 30 nt were obtained from each library. Based on the sequence conservation of mature miRNAs, 418 and 341 conserved miRNAs were identified in ‘Jinnan Shiqin’ and ‘Ventura’ libraries, respectively. Most conserved miRNAs showed high sequence similarities to other plants, and were distributed in 37 conserved miRNA families. Axtell et al. [33] concluded that miRNA families were highly conserved in various plants, and that these miRNAs performed analogous regulatory functions. Some highly conserved miRNA families, including miR156, miR159, miR166 and miR396, showed relatively high numbers of reads in the two celery varieties. Several studies have reported that these miRNAs have crucial roles in biotic and abiotic stress responses, plant development and fertility, and cell proliferation [14,34-36]. Other families, such as miR395 and miR812, had very low abundance in both celery varieties. These findings suggest that over long evolutionary timescales, the miRNAs evolved at different rates and have different roles in plant development. Putative targets gene were predicted to provide more information concerning the identified miRNAs, in which most target genes were involved in molecular functions and transcription pathways.

After searching for potential pre-miRNAs and predicting their hairpin-like structures, 13 and five novel miRNAs were identified in ‘Jinnan Shiqin’ and ‘Ventura’, respectively. Surprisingly, among all the newly identified miRNAs, only one (celery-miR-27) was present in both varieties. Therefore, the functions of these novel celery miRNAs should be analyzed in future studies.

Significantly different abundance was seen for 221 miRNAs between ‘Jinnan Shiqin’ and ‘Ventura’. Different miRNA genes accounted for a large proportion of all identified miRNAs. The two varieties have similar phenotypes, although they originate from different geographical areas: ‘Jinnan Shiqin’ from Asia and ‘Ventura’ from North America. This finding suggests that miRNA evolution in the same plant species is more conservative, and that variations in miRNA machinery in the two celery varieties may be critical for the differences in miRNA expression.

Considering the instability of the environment, plants are frequently challenged by various biotic and abiotic stresses, including temperature stress. Numerous studies have confirmed that small RNAs have important roles in biotic and abiotic stress responses in plants. Some miRNAs are temperature sensitive [37]. For instance, in wheat, miR393 and Ta-miR2002 were up-regulated after 0.5 h of heat treatment [38], while ten cold-regulated miRNAs were detected in *Arabidopsis* by microarray [39]. In castor bean, 41 miRNAs were down-regulated and four others were up-regulated under cold stress compared with normal growth conditions [40]. In the present study, qRT-PCR method was used to investigate six miRNAs under cold and heat stress conditions. All miRNAs in both celery varieties showed sensitivity to temperature change, and abundance was remarkably up-regulated. This finding is consistent with results in *Arabidopsis*, poplar, and wheat [38,39,41]. The stronger response of miRNAs to cold compared to heat stress may be related to celery being a cool-season biennial plant. The abundance of most miRNAs was higher in ‘Jinnan Shiqin’ than in ‘Ventura’, suggesting that ‘Jinnan Shiqin’ is more resistant to temperature stress. This result is consistent with the phenotypes of the two plants in relation to temperature stress. Thus, these miRNAs may have important roles in temperature stress defense. However, understanding the molecular mechanisms underlying these roles requires further study.

## Conclusion

This is the first report to investigate small RNAs in celery; a large number of small RNAs were characterized as known and novel miRNAs. Putative target genes were predicted and then annotated by GO, COG, and KEGG databases to explore gene functions. The differential expression of miRNAs under temperature stress conditions between the two varieties suggests that the miRNA machinery in the two celery varieties may be different. Taken together, this study provides useful information for understanding the functions and regulatory mechanisms of miRNAs.

## Methods

### Plant materials

Celery plants (*A. graveolens* L. cvs. ‘Jinnan Shiqin’ and ‘Ventura’) were grown in pots containing soil:vermiculite mixture (3:1) in a controlled-environment growth chamber programmed for 16 h/8 h at 25°C/16°C for day/night and 3000 lux of light intensity. Two month-old plants were transferred to growth chambers set at 4°C or 38°C under the same light intensity and day length as above. Leaves and stems were collected 1 h after transfer to the hot or cold chambers, immediately frozen in liquid nitrogen, and then stored at −80°C until use.

### RNA isolation, small RNA library development and Solexa sequencing

Total RNA was extracted using an RNA kit (RNAsimple Total RNA Kit, Tiangen, Beijing, China) according to the manufacturer’s instructions. RNA quantity and quality were examined using gel electrophoresis and a Nanodrop ND1000 spectrophotometer. Small RNAs of 18 to 30 nt were separated from total RNAs by polyacrylamide gel electrophoresis. Small RNA molecules were then ligated to Solexa adaptors at both 5’- and 3’-ends, and then converted to cDNA by RT-PCR. The purified DNA samples were sequenced using an Illumina Cluster Station and Illumina Genome Analyzer. The data were submitted to the National Center for Biotechnology Information under accession number, SRR1125031.

### Prediction of conserved and novel miRNAs

After removing the impure sequences (the low quality reads, adaptor reads, and reads with length < 16 or length > 30), unique reads were queried against ribosomal RNAs (rRNAs) and transfer RNAs (tRNAs) from GenBank (http://www.ncbi.nlm.nih.gov/). rRNA, tRNA, small nucleolar RNA (snoRNA), and small nuclear RNA (snRNA) sequences were obtained from Rfam (http://rfam.sanger.ac.uk). Unique reads were also used for a nucleotide–nucleotide Basic Local Alignment Search Tool (BLASTn) search against the miRNA database (miRBase 16.0) to identify conserved miRNAs. To identify novel miRNAs, the Mireap program was used to obtain all candidate precursors with hairpin-like structures that were perfectly mapped by sequencing tags (http://sourceforge.net/projects/mireap). The secondary structures of putative pre-miRNAs were checked using Mfold [42]. The criteria chosen for stem-loop hairpins were described by Meyers et al. [43].

### Prediction of potential miRNA target genes

Target genes of miRNAs were predicted using the psRNA Target program (http://plantgrn.noble.org/psRNATarget). The rules used for target prediction were based on those suggested by Allen et al. [44] and Schwab et al. [45]. BLASTn hits with < 4 mismatches were chosen as candidate targets, and then nucleotide 6-frame translation-protein (blastx) was used to obtain their putative functions.

### Functional annotation of the potential miRNA target genes

To investigate the putative functions of potential target genes, the target sequences were annotated using diverse protein databases, including Gene Ontology (GO), Cluster of Orthologous Groups (COG) and Kyoto Encyclopedia of Genes and Genomes (KEGG) [30,31,46]. The GO categorization results are expressed as three independent hierarchies for biological processes, cellular components, and molecular functions.

### Differential expression of miRNAs between celery varieties

To select differentially expressed miRNAs between the two libraries, the frequency of miRNAs was normalized to one million of the total number of miRNA reads in each sample. The selection method used was according to Audic and Claverie [47]. IDEG6 software [48] was used to analyze the differential expression.

### qRT-PCR analysis of miRNA abundance under temperature stress conditions

Total RNA was extracted using an RNAsimple Total RNA Kit (Tiangen) according to the manufacturer’s instructions. Small RNAs were reverse-transcribed into cDNA using the One Step PrimeScript® miRNA cDNA Synthesis Kit (TaKaRa, Dalian, China). Quantitative real-time PCR (qRT-PCR) was performed using the MyiQ single-color real-time PCR detection system (Bio-Rad, Hercules, CA, USA). The reactions were carried out in a total volume of 20 μL containing 2.0 μL of diluted cDNA, 0.8 μL of each primer, and 10 μL of SYBR GreenI Mix with the following cycling profile: 95°C for 30 s; followed by 40 cycles at 95°C for 5 s, 60°C for 20 s; Melting curve analysis was performed (61 cycles at 65°C for 10 s) to verify specific amplification. Each sample was processed in triplicate, and 5.8S rRNA was used as an internal control. The qRT-PCR primers are listed in Additional file 2: Table S2.

## Competing interests

The authors declare that they have no competing interests.

## Authors’ contributions

ASX conceived and designed the experiments. MYL, FW, ZSX, QJ, GFT and JM performed the experiments. MYL, FW, JQ and ASX analyzed the data. ASX contributed reagents/materials/analysis tools. MYL wrote the paper. MYL and ASX revised the paper. All authors read and approved the final version of manuscript.

## Supplementary Material

Additional file 1: Table S1 The expression profiles of 221 differentially expressed miRNAs. Click here for file

Additional file 2: Table S2 qRT-PCR primer sequences. Click here for file
